# Placenta percreta after Strassman metroplasty of complete bicornuate uterus: a case report

**DOI:** 10.1186/s12884-021-03540-y

**Published:** 2021-01-29

**Authors:** Chengyan Zhang, Xiaoxin Wang, Haili Jiang, Lei Hou, Liying Zou

**Affiliations:** Beijing Obstetrics & Gynecology Hospital, Capital Medical University, 100026 Beijing, PR China

**Keywords:** Bicornuate uterus, Strassman metroplasty, Placenta percreta, Postpartum hemorrhage, Uterine atony

## Abstract

**Background:**

A bicornuate uterus often results in infertility. While reconstructive procedures may facilitate pregnancy, spontaneous abortion or serious pregnancy complications may occur. We present a case of a bicornuate uterus with spontaneous conception after Strassman metroplasty; however, life-threatening complications during pregnancy occurred.

**Case presentation:**

: A 38-year-old woman with a history of infertility presented for prenatal care at 6 weeks of gestation. She had conceived spontaneously after four failed *in vitro* fertilization and embryo transfer (IVF-ET) procedures, Strassman metroplasty for a complete bicornuate uterus, and two postoperative IVF-ET pregnancies that ended in embryo arrest. This pregnancy was uneventful until the patient presented with massive vaginal bleeding at 28 weeks of gestation and was diagnosed with placenta previa and placenta percreta. Bleeding was controlled after emergency Caesarean section and delivery of a healthy neonate. However, severe adhesions were noted as well as a rupture along the metroplasty scar. Two days later, on removal of the intrauterine gauze packing, severe hemorrhage resumed, and the uterus did not respond to oxytocin, hemabate, or carbetocin. Emergency hysterectomy was required.

**Conclusions:**

Reconstructive surgical procedures for complete bicornuate uterus may allow patients to achieve spontaneous pregnancies. However, potential intrapartum complications include placenta implantation and postpartum hemorrhage, and the latter may be exacerbated as the uterus does not contract or respond to oxytocin or prostaglandin drugs. Patients should be counseled on the risks associated with pregnancy after Strassman metroplasty, and clinicians must be aware of potential severe complications.

## Background

The incidence of congenital uterine anomalies is approximately 6.7% in the general population, 7.3% in the infertile population, and 16.7% in the recurrent pregnancy loss population [1]. A bicornuate uterus, which results from incomplete lateral fusion of the two müllerian ducts, accounts for 10–25% of all congenital uterine anomalies [2]. It is associated with obstetric complications including infertility, pregnancy loss during the first and second trimester, preterm labor, intrauterine growth restriction (IUGR), malpresentation, placental abruption, retained placenta, uterine torsion, and spontaneous rupture [3–5]. The more severe the classification and the level of bicornuate uterus, the higher the chance of poor pregnancy outcomes. Surgical intervention with Strassman metroplasty might improve reproductive outcomes for these patients, but there are few reports of severe complications [3]. Incision into the uterine cavity increases the chance of placenta previa, morbidly adherent placenta, and severe postpartum hemorrhage. The latter complication can be lethal, as the malformed and scarred uterus may not contract and respond to oxytocin or prostaglandin drugs. We report a rare case of a complete bicornuate uterus. The patient conceived spontaneously after a Strassman metroplasty procedure, but this was complicated by placenta previa and placenta percreta. The CARE guidelines were followed for this case report [6].

## Case presentation

A 38-year-old woman with a history of having undergone Strassman metroplasty of a complete bicornuate uterus began regular prenatal care in Beijing Obstetrics and Gynecology Hospital, Capital Medical University from 6 weeks of gestation. She had been diagnosed with a bicornuate uterus, endometriosis, and teratoma during laparoscopic ovarian cystectomy for infertility 6 years ago. She had reported no other symptoms related to these conditions. After four failed *in vitro* fertilization and embryo transfer (IVF-ET) attempts due to an inaccessible uterine cavity, she underwent combined hysteroscopic and laparoscopic Strassman metroplasty four years ago. The procedure was converted to open surgery because of dense adhesions. A transverse myometrial incision was made into the uterine cavities from one cornua to the other. The two uterine halves were then sutured vertically from the anterior uterus across the midline fundus and down the posterior wall, creating one unified cavity. Over the next two years, the patient conceived twice by IVF-ET, and both pregnancies ended by curettage for embryo arrest without a chromosomal disorder at 11 weeks of gestation.

**Fig. 1:**
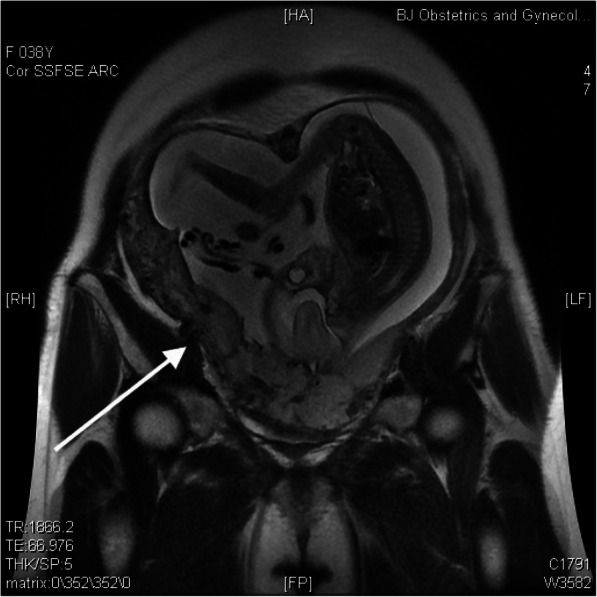
Magnetic resonance imaging at 22 weeks of gestation. Magnetic resonance imaging (MRI) shows the fetus in the bicornuate uterus after Strassman metroplasty, with loss of continuity of the uterine wall and intraplacental bands (white arrow)

**Fig. 2:**
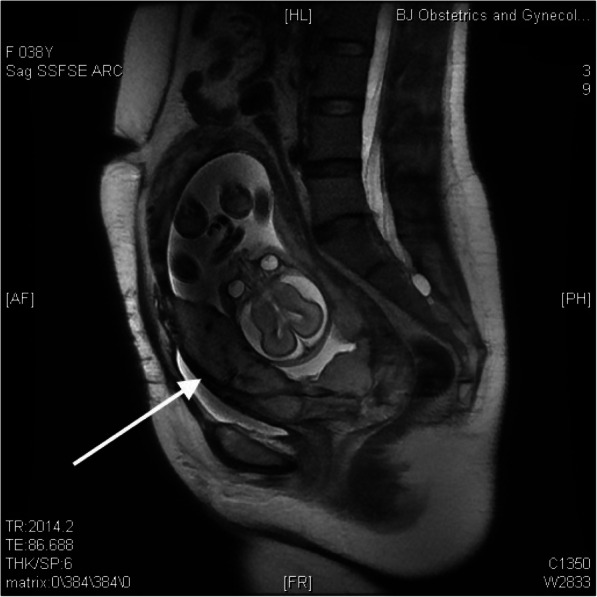
Magnetic resonance imaging at 22 weeks of gestation. Magnetic resonance imaging (MRI) shows the fetus and placenta increta (white arrow)

This pregnancy was naturally conceived, and the patient was under regular antenatal follow-up without the administration of progesterone. Her nuchal translucency scan was normal, and noninvasive prenatal testing indicated low risk. Magnetic resonance imaging (MRI) and ultrasound were suggestive of a posterior-anterior adherent placenta reaching the serosa with anterior myometrial thinning and partially missing at 22 weeks of gestation (Figs. 1 and 2). She reported occasional vaginal bleeding.

At 28 weeks of gestation, the patient went to the emergency room for massive vaginal bleeding after defecating. On arrival, she had lost approximately 600 ml of blood without experiencing any abdominal pain, and there was still active bleeding greater than the amount typical of menstruation. Her pulse rate was 80/min and blood pressure was 110/70 mmHg. Rapid infusion and emergency cesarean section were carried out. During the operation, we noticed severe adhesions around the fragile saddle-shaped uterus. A large number of tortuous vessels covered the lower anterior wall of the uterus. The lower anterior uterine wall adhered to the bladder and was extremely thin, and the posterior wall had closely adhered to the pelvis.

The cesarean section incision was located in the uterine body above the upper edge of the placenta. A healthy boy weighing 1,350 g and measuring 35 cm in height, was delivered from transverse to breech position with a normal cord blood gas analysis and Apgar scores (1, 5, and 10 minutes) of 10-10-10. After the delivery, a 5-cm vertical rupture of the uterus was noticed along the previous metroplasty scar and crossing the transverse incision of the uterus. The tissue beside the scar was much thinner than the surrounding wall. The placenta was on the posterior wall, covered the cervix, and reached the anterior wall. It covered about 4/5 of the uterine cavity and closely adhered to the uterine wall. The patient had severe bleeding after delivery, and 20 IU of oxytocin and 250 µg of carboprost tromethamine were injected into her myometrium to promote uterine contraction. The placenta adhered tightly to the lower uterus and had to be separated manually. A 5 × 6-cm section of the placenta was implanted in the right anterior wall of the uterus and could not be removed. The uterine cavity was filled with gauze and active bleeding was stopped. In addition to the 600 ml hemorrhage before the cesarean, the patient lost another 3,400 ml of blood due to hemorrhage after surgery. The patient was transfused with 10 U of red blood cells, 1,200 ml of plasma, and 1 U of platelets. Subsequently, antibiotics were administered to prevent infection. Over the next 48 hours, the patient had no fever or bleeding, and her vital signs were stable.

Massive vaginal bleeding resumed when the gauze was removed under intravenous anesthesia 48 hours after the Caesarean section, with about 2,000 ml of blood loss in only 5 minutes. Meanwhile, the uterus did not respond to massage or oxytocin, hemabate, or carbetocin administration.

**Fig. 3:**
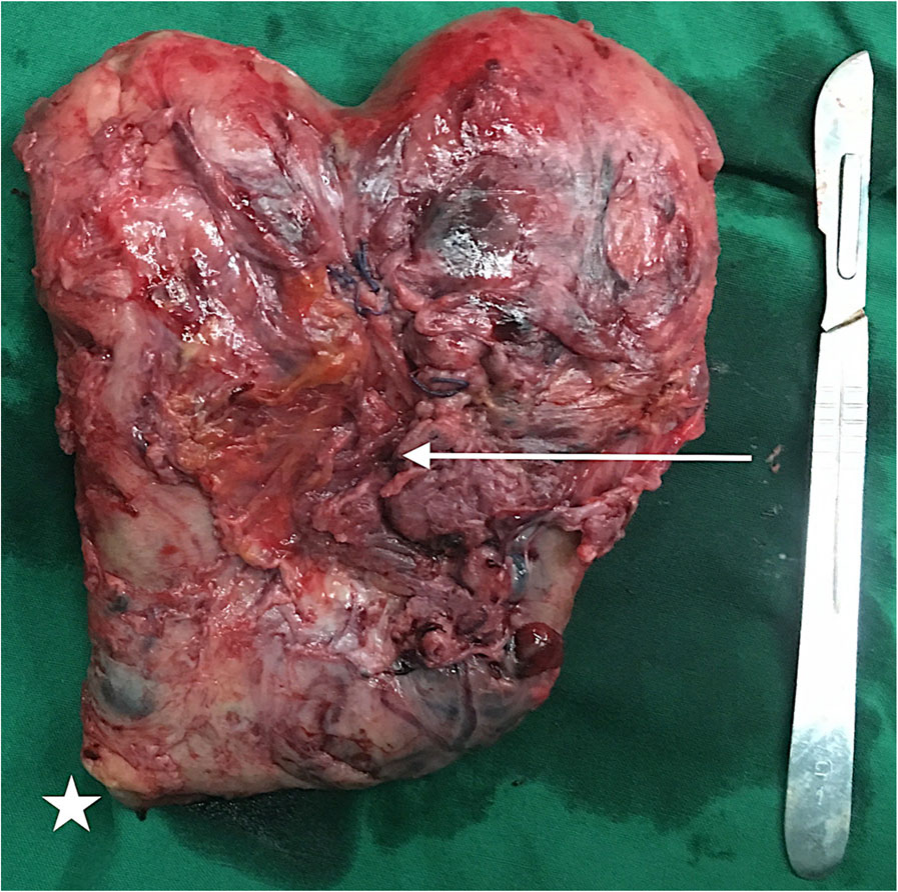
Hysterectomy specimen. The uterus is rigid and non-elastic, especially in the scar area. The longitudinal scar of the fused uterus is 2 mm thick. The placenta adherent to the anterior wall has breached the serosa and adhered to the bladder (white arrow). (White star: cervix)

A transabdominal hysterectomy was performed immediately. The uterus adhered closely to the ovary, intestine, and bladder, and was a rigid saddle shape and bloated to 16 weeks in pregnancy size with blood clots inside (Fig. 3). After the hysterectomy, a 2 × 2-cm rupture was found on the bladder, which was repaired and required a left ureteral stent. The patient lost 2,000 ml and 2,500 ml of blood, respectively, before and during the 5-hour surgery and was transfused with 13 U of RBC, 1,200 ml of plasma, and 1 U of platelets. The postoperative period was uneventful, including bladder irrigation and the administration of intravenous ceftriaxone 2 g once daily for 5 days. The patient was discharged after the ureteral stent extraction. Both mother and child remained well at follow-up 2 years after delivery.

## Discussion and conclusions

According to the Revised American Fertility Society classification, a bicornuate uterus is defined as a double uterus with a single cervix, resulting from incomplete lateral fusion of the two müllerian ducts. The bicornuate uterus is defined as partial or complete according to the degree of division in the uterine corpus [7]. Surgical intervention is not recommended unless the patient has experienced repeatedly abnormal pregnancy outcomes (such as abortion, preterm birth, and infertility) without other causes [8]. The Strassman metroplasty joins the two narrow uterine corpora into one and tries to reconstruct the normal anatomical structure.

Both abdominal and laparoscopic metroplasty can improve uterine morphology, enlarge cavity volume, reduce intrauterine pressure, and increase blood flow to the endometrium and muscle. After surgery, the patient is more likely to achieve conception, and the risk of abortion and preterm delivery is reduced. The fertility and live birth rate associated with metroplasty could reach 70–80% [9, 10]. However, there are reports of less satisfactory prognoses for patients who underwent Strassman metroplasty for a complete bicornuate uterus. In a study of 11 metroplasty patients, the 4 patients with a partial bicornuate uterus all had successful pregnancies, while none of the 7 patients with a complete bicornuate uterus achieved conception [11]. In another report of 10 patients with metroplasty for bicornuate uterus, 4 patients had 5 babies, 3 remained infertile (including 1 case due to male infertility), and 2 were lost to follow-up. One patient had placenta previa and lost 2,000 ml of blood during Caesarean section [12]. No cases of uterine rupture or other intrapartum complications were reported [13].

A bicornuate uterus can cause reduced muscle tissue, abnormal blood flow, and cervical incompetence, all of which may lead to infertility, abortion, preterm delivery, IUGR, and malpresentation [14]. A meta-analysis in 2014 found that a bicornuate uterus was irrelevant to the fertilization rate and the success rate of assisted reproductive technology [15].

In this case, the patient was diagnosed with a complete bicornuate uterus based on findings of laparoscopy combined with hysteroscopic exploration. The abnormal uterus, endometriosis, and pelvic adhesions contributed to infertility and recurrent failed assisted reproductive technology. Abortion in the first trimester was probably caused by inadequate trophoblast blood vessel formation, which was related to the reduced muscle tissue and abnormal vasculature in the process of implantation [16], which might also have led to the lethal placenta percreta [17]. Alternatively, the placenta previa and percreta might have been caused by scar tissue, which formed during metroplasty and curettage. In Xia’s report [12], a similar patient underwent laparoscopic Strassman metroplasty of a complete bicornuate uterus. Two years later, she lost 2,000 ml of blood during surgical delivery due to placenta previa at 37 weeks of gestation. A malformed uterus is usually combined with limited cavity volume, less elastic muscle, and cervical incompetence, which are relevant to abortion, preterm delivery, and malpresentation.

Despite Strassman metroplasty, the uterus, in this case, was still morphologically abnormal, and the horns did not expand during pregnancy. Limited cavity volume is likely the reason for the massive vaginal bleeding and preterm delivery. Although we did not expect uterine rupture and preterm birth in our patient and did not, therefore, implement measures to extend her pregnancy or prevent uterine rupture, we believe that her prognosis might have been improved by cervical cerclage at 12 to 14 weeks of gestation. We only scheduled the patient’s hospitalization at around 28 weeks of gestation for administration of antenatal corticosteroids to enhance fetal lung maturation and multidisciplinary consultation with the neonatology, anesthesia, and intervention departments for the surgical planning. However, prior to this scheduled appointment, our patient experienced massive vaginal bleeding, which did not allow us to conduct the intended multidisciplinary consultation and led us to perform a hysterectomy. The postpartum hemorrhage after gauze removal probably occurred because the uterus did not contract and respond to oxytocin or prostaglandin drugs. In the normal uterus, active hemorrhage seldom occurs after 48-hours of compression. However, patients with a malformed uterus have a high risk of massive bleeding during gauze removal because of muscle hypoplasia, which is complicated by poor vessel contraction and does not respond to massage and contraction drugs. Recently, there were two cases reported with successful management of postpartum hemorrhage in a bicornuate uterus with balloon tamponade and no massive bleeding on balloon removal [18, 19]. Therefore, tamponade of the uterine cavity may be effective in the management of postpartum hemorrhage in the presence of a bicornuate uterus. Due to the high bleeding risks, uterine artery embolization is recommended for these patients after Caesarean section and uterine packing, and insertion of an aortic sac and ureteral stent before gauze removal may also be recommended. Although we were unable to utilize any of these methods on our patient, in cases such as this one, measures such as uterine artery embolization, insertion of an aortic sac before gauze removal, and balloon tamponade, might reduce vaginal bleeding and avoid hysterectomy after gauze removal.

Regular antenatal care, color flow ultrasound, and MRI may be needed for the evaluation of an adherent placenta in patients with a history of Strassman metroplasty. Further research is needed to evaluate the optimal methods of treating postpartum hemorrhage with congenital uterine anomalies.

Women with a bicornuate uterus must be counseled on the severe complications that may occur during pregnancy after Strassman metroplasty, which can result in hysterectomy. Further, a scheduled Caesarean section is the recommended mode of delivery due to the risk of uterine rupture [20]. Clinicians must monitor patients carefully for intrapartum complications including placenta implantation and postpartum hemorrhage.

## Data Availability

All data generated or analyzed during this study are included in this published article.

## Abbreviations

IVF-ET: In vitro fertilization and embryo transplantation
IUGR: Intrauterine growth restriction
MRI: Magnetic resonance imaging
